# Developing a new generation of breast cancer clinical gene expression tests

**DOI:** 10.1186/bcr3688

**Published:** 2014-07-07

**Authors:** Zuzana Kos, Torsten O Nielsen

**Affiliations:** 1 Department of Laboratory Medicine and Pathobiology, University of Toronto, 1 King's College Circle, Toronto, ON M5S 1A8, Canada; 2 Genetic Pathology Evaluation Centre, Department of Pathology and Laboratory Medicine, University of British Columbia, 2660 Oak Street, Vancouver, BC V6H 3Z6, Canada

## Abstract

When treatment decisions are based purely on clinicopathological factors, many women with estrogen receptor-positive/human epidermal growth factor receptor 2-negative cancers are overtreated. Gene expression profiles are valuable clinical tools that stratify the recurrence risk to identify patients most likely to benefit from adjuvant systemic therapies. Building upon greater understanding of tumor biology and more rigorous approaches to validation (including independent studies with a high level of evidence), several second-generation multigene tests have been developed. In the previous issue, Martin and colleagues report the third clinical validation study for EndoPredict, a distributed assay to assess risk of distant recurrences in estrogen receptor-positive/human epidermal growth factor receptor 2-negative women. The authors confirm the assay’s independent prognostic value in premenopausal and postmenopausal, node-positive women treated with contemporary chemotherapy followed by endocrine therapy. EndoPredict did not, however, predict benefit from adding paclitaxel. Predictive signatures for selecting among chemotherapy regimens remain an area needing further development.

## 

When treatment is based solely on clinicopathological risk factors, many women with estrogen receptor-positive/human epidermal growth factor receptor 2-negative (ER^+^/HER2^−^) tumors are overtreated – subjected to morbidity from cytotoxic chemotherapy for negligible benefit. Identifying patients safely treated by endocrine therapy alone has driven the development of prognostic gene expression assays.

In the previous issue, Martin and colleagues describe the third clinical validation of EndoPredict (EP; Sividon Diagnostics GmbH, Cologne, Germany) [1], a second-generation multigene test trained to predict distant recurrence in ER^+^/HER2^−^ tumors, and by extension the need for adjuvant chemotherapy. EP was previously validated in prospective–retrospective analyses of endocrine-treated postmenopausal ER^+^/HER2^−^ breast cancer patients in two clinical trials (ABCSG-6 and ABCSG-8) [2].

Genomic-based assays developed from complex, high-dimensional data are susceptible to overfitting. Clinical validation must be performed in entirely independent datasets using predefined, locked-down classifier algorithms and analysis plans. Martin and colleagues’ study exemplifies the rigor required by Simon and colleagues for a formal prospective–retrospective study to contribute to generating level IB evidence [3]. The authors present EP validation results in ER^+^/HER2^−^ patients from the GEICAM/9906 clinical trial of node-positive women treated with contemporary chemotherapy. The prognostic ability of EP remains robust in this higher risk group, identifying a 10-year metastatic rate of 7% in the predefined EP low-risk group (versus 30% in the high-risk group). Including tumor size and nodal status as the EPclin classifier identifies a small (13%) but impressively low-risk cohort of women who experienced no distant recurrences at 10 years. As the whole of this cohort received chemotherapy, the clinical utility of this finding (to avoid chemotherapy) is difficult to infer, although the 100% metastasis-free survival in both the fluorouracil–epirubicin–cyclophosphamide and the fluorouracil–epirubicin–cyclophosphamide–paclitaxel randomized arms does imply no benefit from adding paclitaxel.

The authors speculate that the EPclin low-risk group (recurrence-free at 10 years) in this 5-year endocrine therapy-treated population may identify women not needing extended endocrine therapy, consistent with the ABCSG-8 trial [4]. This promising idea must be interpreted cautiously given that only 16 of the 74 EPclin low-risk patients had 10-year follow-up data. Both the aTTom and ATLAS trials have shown that survival benefits of extended hormonal therapy become more apparent after year 10 [5,6].

Several multigene prognostic assays have now been developed for use in ER^+^/HER2^−^ breast cancers. First-generation assays including MammaPrint (MammaPrint: Agendia, Amsterdam, The Netherlands) and Onco*type* DX (Oncotype: Genomic Health, Redwood City, CA, USA) suffered from early methodological issues, most seriously a failure to maintain rigorous separation between training and validation sets, and inclusion of nonluminal and/or HER2^+^ tumors in their training sets, thereby allowing these high-risk tumors to skew outcome-related gene selection away from the relevant patient group [7-9]. MammaPrint was specifically trained around early relapse (within 5 years) in node-negative women, most having received no adjuvant systemic therapy, and has not been shown to predict late recurrence outside the original training-validation cohort. Onco*type* DX heavily weighed the tamoxifen-only arm of the NSABP-B20 trial in its training set, where most recurrences occurred within 5 years, and has diminished prognostic ability beyond year 5 [10].

More recently, building upon biological and technical advances and more rigorous approaches to validation, second-generation multigene tests have been developed, including the Breast Cancer Index (BCI: bioTheranostics, San Diego, CA, USA), PAM50 (PAM50: NanoString Technologies Inc., Seattle, WA, USA) and EP. The Breast Cancer Index combines a molecular grade index (quantifying tumor grade-associated genes) and a two-gene ratio, *HOXB13*:*IL17BR*, related to estrogen signaling [11]. PAM50, unlike signatures trained around outcome, was developed as a biological classifier of the major intrinsic molecular subtypes of breast cancer [12]. These three assays predict both early and late recurrences [4,10,13].

IHC4 and Mammostrat (Mammostrat: Clarient, Inc., Aliso Viejo, CA, USA) immunohistochemical panels are also prognostic in early breast cancer [14,15]. IHC4 uses standard pathology markers (ER, progesterone receptor, HER2 and Ki67) to provide prognostic information comparable with Onco*type* DX [14]. Immunohistochemical staining and scoring does suffer from limited analytical reproducibility, probably contributing to Martin and colleagues’ identification of low Ki67 scores (<14%; a published cutoff point for good-prognosis luminal A tumors) in a surprisingly high fraction (almost three-quarters) of this node-positive cohort [16].

Each of these gene expression and immunohistochemical panels identifies a good prognosis group that may not need chemotherapy. Emerging evidence suggests that some panels identify women at such low risk of late recurrence that they may safely avoid extended endocrine therapy. For high-risk women, however, the question is not one of chemotherapy versus no chemotherapy, but rather a question of which chemotherapy agent(s) will be most effective for which patients – a true predictive indication. In Martin and colleagues’ report, the EP score did not predict benefit from adding weekly paclitaxel to fluorouracil–epirubicin–cyclophosphamide chemotherapy. Outcome-trained signatures from nonchemotherapy populations are unlikely to predict between chemotherapy regimens; Table 1 summarizes some relevant features of the referenced molecular signatures, including predictive studies.

**Table 1:** Overview of selected multigene signatures for breast cancer

**Assay; platform, clinical material**	**Training parameter**	**Approval or endorsement**	**Analytical validity: published assay validation**	**Clinical validity: prognosis validation**	**Predicting treatment benefit using randomized clinical trials**					**Randomized prospective trials**
					**Tamoxifen**	**Herceptin**	**Chemotherapy versus no chemotherapy**	**Specific agent: anthracycline**	**Specific agent: taxane**	
Breast Cancer Index; RT-PCR, FFPE (central)	Outcome (ER^+^, pN0, endocrine-treated women) MGI component – biology (tumor grade related genes) H:I component – outcome (recurrence in tamoxifen-treated women)	No	No	ATAC [13], Stockholm [17], multiple nonrandomized trial cohorts	No^a^	No	No	No	No	No
EndoPredict; RT-PCR, FFPE (distributed)	Outcome (distant recurrence in endocrine-treated ER^+^/HER2^−^ pN0/pN^+^ women)	CE Mark	Yes [18,19]	ABCSG6 [2], ABCSG8 [2], GEICAM/9906 [1]	No	No	No	No	GEICAM/9906 [1] (failed to predict benefit)	No
IHC4; IHC, FFPE (distributed)	Outcome (distant recurrence in ER^+^ endocrine-treated women)	No	No	ATAC [14], TEAM [20]	No	No	No	No	No	No
MammaPrint; microarray, fresh and FFPE (central)	Outcome (5-year metastasis rate in pN0 women)	FDA (fresh): risk for distant metastasis, <61 years, stage I and II, tumor ≤5 cm and node-negative	No	Multiple nonrandomized trial cohorts including RASTER	No	No	No	No	No	MINDACT prognosis validation (to report 2015)
Mammostrat; IHC, FFPE (central)	Outcome (unselected cohort of breast cancer patients)	No	No	NSABP-B14, NSABP-B20 [15] multiple nonrandomized trial cohorts	No	No	NSABP-B20 (±CMF) [15] (all women benefit – high risk benefit the most)	No	No	No
Onco*type* DX; RT-PCR, FFPE (central)	Outcome (recurrence in mainly tamoxifen-treated ER^+^, pN0 women)	NCCN, ASCO, St. Gallen (role for identifying women that may benefit from chemotherapy)	Yes [21]	NSABP-B14 [9], NSABP-B28 [22], SWOG8814 [23], multiple nonrandomized trial cohorts	NSABP-B14 [24] (largest benefit in quantitative ER high/recurrence risk low patients)	No	NSABP-B20 (±CMF) [25], SWOG8814 (±CAF) [23] (benefit from chemotherapy with high recurrence score)	No	NSABP-B28 [22] (failed to predict a benefit)	TAILORx (node-negative, to report 2015) RxPONDER (one to three positive nodes, recruiting)
PAM50 (research based assay); RT-PCR and microarray, FFPE and fresh (distributed)	Biology (identification of major molecular subtypes)	N/A research assay	No	NCIC-MA5 [26], NCIC-MA12 [27], GEICAM/9906 [28], multiple nonrandomized trial cohorts	NCIC-MA12 [27] (luminal subtype predicts benefit)	NOAH [29] (HER2-enriched benefits the most)	No	NCIC-MA5 [26] (CMF vs. CEF; epirubicin benefit in HER2-enriched subtype only)	GEICAM/9906, CALGB/9342 and CALGB/9840 [30] (low proliferation score predicts weekly paclitaxel benefit)	No
Prosigna; nCounter, FFPE (distributed)	Biology (subtype); outcome (ROR score)	CE Mark, Health Canada, FDA: prediction of 10-year DRFS in ER^+^, node 0 to 3, postmenopausal women treated with endocrine therapy	Yes [31]	ATAC [32], ABCSG08 [33]	No	No	No	No	No	RxPONDER (one to three nodes, recruiting; embedded additional analysis)

CAF, cyclophosphamide, doxorubicin, fluorouracil; CEF, cyclophosphamide, epirubicin, fluorouracil; CMF, cyclophosphamide, methotrexate and fluorouracil; DRFS, distant relapse-free survival; ER, estrogen receptor; FDA, US Food and Drug Administration; FFPE, formalin-fixed paraffin-embedded; HER2, human epidermal growth factor receptor 2; H:I, *HOXB13*:*IL17BR*; IHC, immunohistochemistry; MGI, molecular grade index; N/A, not applicable; pN0, pathological lymph node-negative; pN^+^, pathological lymph node-positive; ROR, risk of recurrence; RT-PCR, reverse transcription polymerase chain reaction. Breast Cancer Index: bioTheranostics, San Diego, CA, USA; EndoPredict: Sividon Diagnostics GmbH, Cologne, Germany; IHC4: MammaPrint: Agendia, Amsterdam, The Netherlands; Mammostrat: Clarient, Inc., Aliso Viejo, CA, USA; Onco*type*: Genomic Health, Redwood City, CA, USA; PAM50: NanoString Technologies Inc., Seatlle, WA, USA; Prosigna: NanoString Technologies Inc., Seattle, WA, USA. ^a^Nested cohort study using material from NCIC CTG MA.17 – HOXB13/IL17BR predictive of benefit from extended letrozole.

What does the future hold for gene expression signatures? Cheaper and faster next-generation sequencing has been touted as the pinnacle of personalized medicine, destined to render multigene expression assays obsolete. However, the genetic complexity of tumors (copy number variations, chromosome-scale structural changes, thousands of mutations, epigenetic changes and intratumoral genetic heterogeneity) is proving even more complex than anticipated. Much as the increased detail from electron microscopy never did replace light microscopy for cancer diagnosis, the broader signatures detected by representative gene expression profile assays, reflecting clinically significant patterns common across many patients, are likely to remain relevant for important treatment decisions.

### Abbreviations

EP: EndoPredict; ER: Estrogen receptor; HER2: Human epidermal growth factor receptor 2.

### Competing interests

TON reports a proprietary interest in the PAM50 assay, which has been licensed to Nanostring Technologies. ZK has no competing interests.
